# Investigation of Early Supplementation of Nucleotides on the Intestinal Maturation of Weaned Piglets

**DOI:** 10.3390/ani11061489

**Published:** 2021-05-21

**Authors:** Federico Correa, Diana Luise, Ivonne Archetti, Paolo Bosi, Paolo Trevisi

**Affiliations:** 1 Department of Agricultural and Food Sciences (DISTAL), Alma Mater Studiorum-University of Bologna, Viale G. Fanin 46, 40127 Bologna, Italy; federico.correa2@unibo.it (F.C.); diana.luise2@unibo.it (D.L.); paolo.trevisi@unibo.it (P.T.); 2 Istituto Zooprofilattico Sperimentale della Lombardia e dell’Emilia Romagna (IZSLER), “Bruno Ubertini”, Via Bianchi, 9, 25124 Brescia, Italy; ivonne.archetti@izsler.it

**Keywords:** immunity, intestinal maturation, microbiota, nucleotides, piglet, transcriptome

## Abstract

**Simple Summary:**

Nucleotides represent a group of bioactive compounds essential for the development of the gastrointestinal tract and immune function. This study aimed to evaluate the short-term effect of oral administration of nucleotides before and after weaning on growth performance, health, development of the intestinal immunity and microbiome of piglet. A nucleotide-based product (NU) was orally given four times before weaning and once after to one group of piglets, while a second group was used as a control (CO). The NU pigs did not grow more than the control until 12 days post-weaning but had increased hemoglobin and hematocrit values. At weaning, feces of NU piglets had a microbial profile more typical of growing pigs, while those of CO were more representative of suckling pigs. The upregulation of genes in the blood of control pigs at weaning was indicative of more activation towards an inflammatory response, while genes of erythropoiesis were more active in NU pigs post-weaning. NU supplementation stimulated genes for proliferative activity in the intestinal immune system, a sign of possible anticipated maturation. NU supplementation did not influence the growth performance of piglets but may have expressed a positive effect on pig microbiota anticipating its maturation at weaning, with possible immunostimulant activity on the intestinal immune system.

**Abstract:**

Nucleotides are essential for the development of the gastrointestinal tract and immune function, but their intake with milk by piglets could be insufficient. The effect of nucleotides on growth and health was tested on 98 piglets divided into two groups: NU, orally administrated with 4 mL of a nucleotide-based product (SwineMOD^®^) at 10, 15, 18, 21, 27 days, or not (CO). Blood and feces were sampled at weaning (26 d, T1), and at 38 d (T2). Per each group and time-point, eight piglets were slaughtered and jejunal Peyer’s patches (JPPs) were collected. NU increased hemoglobin content and hematocrit, but not growth. At weaning, the NU fecal microbiota was characterized by the abundance of Campylobacteraceae, more typical of the growing phase, compared to CO, with a greater abundance of Streptococcaceae. For the blood transcriptome, an initial greater inflammatory activation was seen in CO, while at T2, NU enriched gene sets related to erythropoiesis. The activation of gene groups ranging from epigenetic response to transcriptional regulation evidenced an intense proliferative activity in NU JPPs. NU supplementation did not influence the growth performance of piglets but could have expressed a positive effect on pig microbiota anticipating its maturation at weaning. This immunostimulant activity in the JPPs could moderate the inflammation in the immediate pre-weaning.

## 1. Introduction

Nucleotides are a group of bioactive compounds representing building units of nucleic acids (DNA and RNA) and are involved in various biochemical processes. They are composed of a nitrogenous base (pyrimidine or purine) bound to a pentose (ribose or deoxyribose) sugar to which one, two or three phosphate groups are connected. They are considered essential for tissue with high cellular turnover [1], such as the intestinal epithelium and lymphoid tissues, that lack de novo synthesis of nucleotides [2] and rely mostly on the salvage pathway that requires an external supply of nitrogenous bases. In some physiological conditions such as weaning, there is a limited nutrient intake and a higher cellular proliferative activity of the gut and immune system that are not fully developed. In these cases, nucleotide supplementation as growth promoters and immune stimulators could be essential in piglets [3,4]. 

In addition, suckling and weaning are, for the piglet, the two most critical phases when it undergoes enteric pathologies that can have detrimental effects on its health, requiring antibiotic and therapeutic interventions. Furthermore, pre-weaning piglets are frequently vaccinated to improve their resistance to the main causes of colibacillosis (Enteropathogenic strains of *Escherichia coli* = ETEC), and this requires an active and mature response of the immune system. Nucleotides have shown potential value to mitigate the effect of weaning on piglet growth and/or health [3,4,5]. There are also some pieces of evidence that nucleotides could favor the development of a healthier microbiota. In pigs, this was by increasing lactobacilli and bifidobacteria and reducing *Clostridum perfringens* counts [6]. In pathogen-free chickens, it was by increasing intestinal bacterial diversity and the abundance of Lactobacillus [7].

During the suckling phase, the main source of nucleotides is represented by the maternal milk, but their concentration in sow milk tends to decrease with the proceeding of lactation [6]. Therefore, it is possible that the contribution to the specific nutritional requirement of the digestive and immune system by the milk nucleotides tends to decrease progressively, thus also delaying the maturation of the digestive system and immunity. This factor can influence the stabilization of the intestinal microbiota and the growth performance in the post-weaning phase. Curiously, most of the studies conducted on nucleotides are focused on the post-weaning phase, not considering the possible supplementation during suckling.

The aim of this study was to evaluate the effect of oral administration of nucleotides before weaning and in the immediate post-weaning on growth performance, health, intestinal structure, immunity, microbiota, and transcriptomic profile of weaned piglet preliminary vaccinated against ETEC.

## 2. Materials and Methods

### 2.1. Experimental Design and Sampling

The trial was carried out in a 300-sow farm that uses a vaccine prophylaxis for diarrhea caused by ETEC expressing F4 and F18 fimbriae. A total of 8 sows from homozygotes susceptible to *E. coli* F4 infection were selected. This was done to obtain all susceptible piglets (homozygotes or heterozygotes, determined by the sire) from these sows, that for this reason were potentially immunologically reactive to the vaccination [8]. For the genotyping, DNA was extracted from the bulbs of bristles obtained from each sow and genotyped for MUC4 g.8227C>G, by restriction fragment length polymorphism PCR (PCR-RFLP) using specific primers [9]. The sows belonged to two temporarily consecutive batches of farrowing. At 10 days of life, a total of 96 piglets (3.5 ± 0.6 kg), balanced by live weight and litter of origin, were randomly assigned into the two experimental groups. In the treatment group (NU = 48; 3.48 ± 0.72 kg body weight), piglets were orally supplemented with 4 mL of a solution containing a total 100 mg of a product standardized in nucleotides (swineMOD^®^, Prosol, Madone -BG-, Italy), and in the control group (CO = 48; 3.52 ± 0.63 kg body weight), pigs were orally supplemented with 4 mL of pure water. The supplements were orally administered using a dosing device at 10, 15, 18, 21 days of life, and 1-day post-weaning. The use of an oral solution was preferred to the integration of the creep feed to ensure an equal intake for all subjects. Creep-feed was available from day 15. The creep-feed ingredients and estimated analytic composition are reported in Table 1.

At weaning (26 days of age, T1), piglets were transferred to the experimental facility of the University of Bologna. There, a total of 16 piglets (8 piglets per group) balanced by body weight and litter were slaughtered. The rest of the piglets were raised for twelve days with the same pre-starter feed used during the suckling period, and at the end of the experimental trial (12 d post-weaning, T2), another 16 piglets (8 piglets per group) derived from the same litters were slaughtered. After weaning, pigs were reared individually, penned inside a weaning room at pre-controlled temperatures and ventilation. The pens (100 cm × 33 cm each) were side-by-side, allowing contacts of the muzzles of neighbor pigs. Feed and water were freely available.

### 2.2. Growth Performance and Samplings

Piglets were weighed individually at 10, 21, and 26 days of age and then at the end of the experimental trial (12 d post-weaning). After weaning, the amount of feed supplied to the piglets was recorded daily and corrected for any residual feed to calculate the average feed intake.

The incidence of diarrhea was assessed as the number of days of diarrhea per pig, considering those with diarrhea as having a fecal score value above 3. The fecal score was defined using a five-point scale from 1 = hard feces to 5 = liquid feces.

For the analysis of the microbial profile, fecal samples at T1 ((16 pigs selected for the slaughtering) and at T2 (80 pigs, including 16 pigs destined to the second slaughtering) were collected. At the same time points, the same piglets were sampled for peripheral blood (PB). Samples were collected from the jugular vein, using BD Vacutainer with EDTA K3 and BD Vacutainer with clot activators for serum collection. Blood was centrifuged at room temperature after 2 h of incubation to obtain serum. On the samples, a hemogram was determined. The hemochromocytometric analysis was performed using the automatic analyzer CELL-DYN 3700R^®^” (Abbott Laboratories; Abbott Park, IL, USA) [9].

The pigs to be slaughtered were chosen based on the average body weight (T1, 7.02 ± 0.81 kg; T2, 9.76 ± 0.57 kg), sedated by anesthesia with Zoletil 100 (15 mg/kg) and slaughtered with an intracardiac injection of Tanax^®^ (0.5 mL/kg). From the same pigs, an additional PB sample was taken before anesthesia for mRNA sequencing. The PB was collected using Tempus™ blood RNA tubes (Thermo Scientific, Waltham, MA, USA) and stored at −80. In addition, a sample of jejunal Peyer’s patches (JPPs) was collected from the distal third part of jejunum, and also for mRNA sequencing. JPPs were selected for testing post-natal immune activation because more involved in the diversification of immunoglobulin production and less in their primary undiversified production than ileal PPs in swine [10]. JPPs were collected in sterile tubes and immediately frozen in liquid nitrogen and stored at −80 °C until processed.

#### Statistical Analysis of Growth Performance and Blood Parameters

Data on body weight (BW), average daily gain (ADG), feed intake (FI), feed to gain ratio (F:G), and hematocrit were analyzed with an ANOVA model considering diet, litter of origin and sex as factors, using GLM procedure of SAS (SAS Inst. Inc., version 9.4, Cary, NC, USA).

### 2.3. Microbiota Profiling

The bacterial DNA extraction was carried out using QIAamp Fast DNA Stool Mini Kit (QIAGEN, Hilden, Germany). The V3-V4 region of the 16S rRNA gene was amplified using Pro341F: 5′-TCGTCGGCAGCGTCAGATGTGTATAAGAGACAGCCTACGGGNBGCASCAG-3′ and Pro805R:5′-GTCTCGTGGGCTCGGAGATGTGTATAAGAGACAGGACTACNVGGGTATCTAATCC-3′ [11], using PlatinumTM Taq DNA Polymerase High Fidelity (Thermo Fisher Scientific, Monza, Italy). The PCR reaction conditions for amplification of DNA were as follows: initial denaturation at 94 °C for 1′, followed by 25 cycles of denaturation at 94 °C for 30″, annealing at 55 °C for 30″ and extension 65 °C for 45″, ending with 1 cycle at 68 °C for 7′. The libraries were prepared using the standard protocol for MiSeq Reagent Kit v3 and were sequenced on the MiSeq platform (Illumina Inc., San Diego, CA, USA). For the bioinformatics analysis, the DADA2 pipeline was used [12] using the Silva database (version 132) as reference for the taxonomic assignment.

#### Statistical Analysis of Microbiota

The statistical analysis on alpha diversity, beta diversity, and taxonomic composition was carried out with R v3.6, using the PhyloSeq [13], Vegan [14] and lme4 [15] packages. An ANOVA and a PERMANOVA (“adonis” procedure) models were used to test the effect of age and treatment, on alpha and beta diversity, respectively. These models were applied on the datasets divided according to the two time points, while the effect of time was tested on the entire data set. The differences in taxonomic composition were tested using the DESeq2 package based on negative binomial generalized linear models (Love et al., 2014).

### 2.4. Transcriptome

#### 2.4.1. Pig mRNA Extraction and Sequencing

For blood samples collected in Tempus™ tubes, total RNA was isolated using the Tempus™ Spin RNA Isolation Kit (Thermo Scientific, Waltham, MA, USA) following the manufacturer’s instructions. For tissue samples, total RNA was extracted using the GeneJET RNA Purification Kit (Thermo Scientific, Waltham, MA, USA) according to the manufacturer’s instructions. DNase treatment was performed to remove contaminating DNA using TURBO DNA-free™ DNA Removal Kit (Thermo Scientific, Waltham, MA, USA) following the recommended protocol. RNA quantity and quality were evaluated using a Nanodrop ND 1000 spectrophotometer (Nanodrop Technologies Inc., Wilmington, DE, USA) and agarose gel electrophoresis, respectively. RNA integrity was evaluated through Agilent Bioanalyzer 2100 (Agilent Technologies, Santa Clara, CA, USA). Libraries were prepared using the TruSeq Stranded mRNA Sample Preparation kit and sequenced using the Illumina MiSeq system 2 × 100 bp with 2 × 20 million sequencing depth.

#### 2.4.2. Differential Expression Analyses of RNA-Seq Data

After quality control using the FastQC tool (v.0.11.9), reads were filtered with Trimmomatic (v.0.36) [16] by trimming leading and trailing bases with a Phred score less than 2 and dropping reads shorter than 15 bases long and those with average Phred score per base less than 15. Sequences were aligned to the NCBI *Sus scrofa* v11.1 reference transcriptome using salmon (v.0.14.1) [17]. Differential expression analysis was carried out in R (3.6.2) using the DESeq2 package (v.1.26) [18], testing for the effect of the diet in the two different time-points separately. Genes were considered differentially expressed (DE) when a p-value adjusted (p adj) for a false discovery rate (FDR) < 0.05 and a Log2FC > 2.

#### 2.4.3. Functional Enrichment Analysis

For the functional enrichment analysis, an exploratory analysis was conducted using the Gene Set Enrichment Analysis (GSEA) software [19], which performs an analysis of gene sets, defined as groups of genes with common biological function, chromosomal position, or regulation. The GSEA analysis was based on the C5 sub-collections of Gene Ontology and on the Hallmark collection [20] (MSigDB, Broad Institute, and UC San Diego), and the gene sets were considered significantly enriched with a q value of FDR ≤ 0.05. Finally, to evaluate the differences between diets by combining either times or tissues, the EnrichmentMap Plugin [21] for Cytoscape 3.8 [22] was used, which displays the overlap and connections between different gene set, considering a q value of FDR <0.05 or <0.001, depending on what is specified later, per each presented enrichment map. The nodes were joined if the overlap coefficient was ≥0.375.

## 3. Results

### 3.1. Growth Performance and Blood Parameters

Table 2 shows the growth performance data. In general, no statistically significant differences were observed for all the parameters analyzed.

For the blood parameters (Table 3), there was no statistically significant interaction between the diet and sampling time-point; consequently, the effect of the two factors was assessed separately and the value for the diet is inclusive of the two sampling times. Pigs treated with nucleotides (NU) had a higher hemoglobin content (12.0 vs. 11.8, *p* < 0.05) and hematocrit percentage (36.9 vs. 35.4, *p* < 0.05) than pigs in the CO group.

### 3.2. Microbiota Profile

The sequencing process produced a total of 3,459,059 reads (69,348 on average). Two samples did not produce enough reads, and thus were removed from the analysis. In total, the number of subjects in the analysis were 93 (46 NU, 47 CO, 16 T1, 77 T2). A total of 8591 amplicons sequences variants (ASVs) were identified, which resulted in 23 different phyla (Firmicutes 42.48%, Bacteroidetes 41.71%), 66 Families (Prevotellaceae 21.55%, Ruminococcaceae 16.91%) and 204 genera (Prevotellaceae_NK3B31_group 7.68%, Rikenellaceae_RC9_gut_group 6.45%).

**Figure 1:**
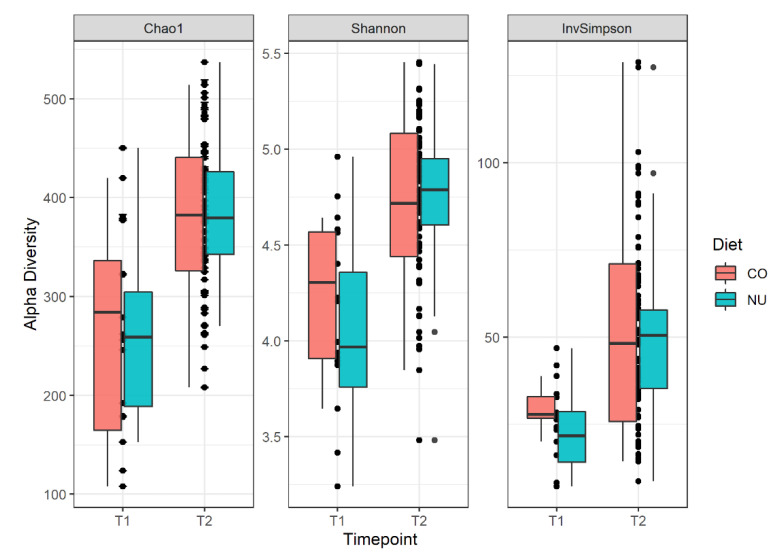
Alpha diversity boxplots with Chao1, Shannon and Simpson indices for the piglets divided into the 2 experimental groups (NU, CO) and the 2 sampling times (T1,T2). Simpson index, *p* = 0.06.

The alpha diversity values (variability within communities), measured with Chao1, Shannon, and Simpson indexes (Figure 1), did not differ between the two diets both at weaning (T1) and at the end of the trial (T2). However, at T1, there was a strong trend for the Simpson index and the CO group had a higher value than NU group (CO: 0.965, NU: 0.937, *p* = 0.06).

**Figure 2:**
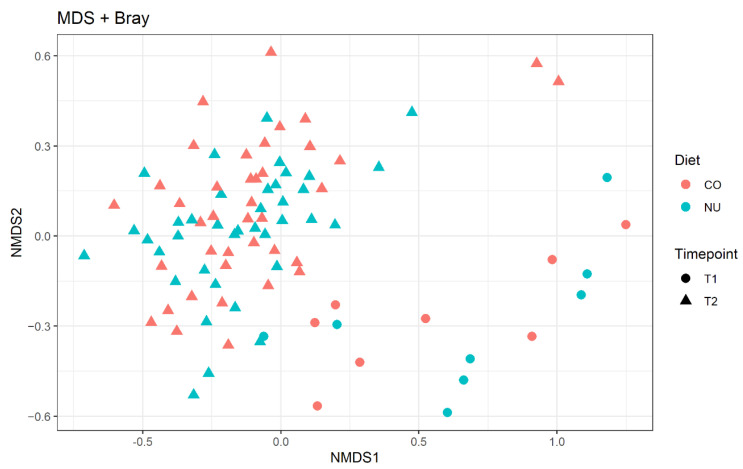
Non-metric multi-dimensional scaling (NMDS) plot on Bray–Curtis distances at ASVs level. CO: control group, NU: nucleotide supplementation. T1: weaning. T2: 12-d post weaning.

For the beta diversity (compositional similarity between microbial communities), the results were plotted using a nonmetric multidimensional scaling plot (NMDS; Figure 2). The effect of age was evident and visible in two distinct clusters and it was confirmed by the Adonis test (R^2^ = 0.06; *p* = 0.01). There were no differences between the two diets at both time-points (T1 and T2).

For the taxonomic composition, at weaning, the phylum Epsilonbacteraeota was significantly more abundant in the NU group compared with the CO group (log2FC: 2.30, *p* adj < 0.01); within this phylum, the most represented family in NU was that of Campylobacteraceae (log2FC: 3.14, *p* adj: 0.03), while Streptococcaceae were more abundant in the CO group (log2FC: —5.36, *p* adj < 0.01). There were no statistically significant differences regarding the taxonomic composition between the two diets at T2.

### 3.3. Transcriptome Profile

**Figure 3:**
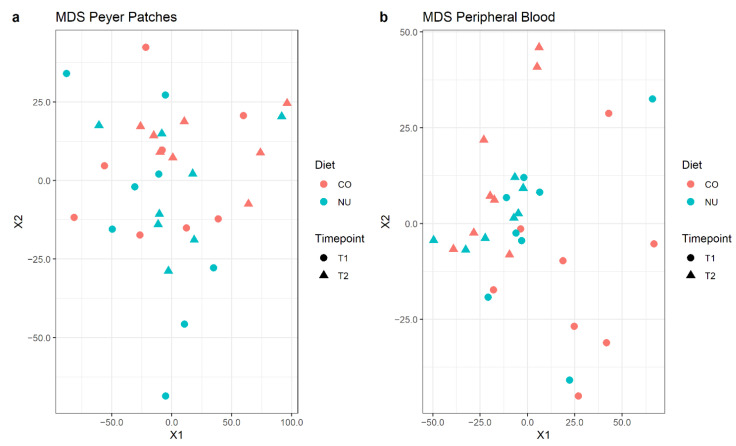
Multi-dimensional scaling (MDS) plots for normalized gene expression levels of JPP (**a**) and PB (**b**) at weaning (T1) and 12 d-post-weaning (T2), in control (CO) and nucleotide (NU) groups.

Transcriptomic analysis was carried out on a total of 32 samples per tissue (PB or JPPs), divided into the two timepoints (weaning or 12 d post-weaning). However, for a single blood sample (NU, T2 group), it was not possible to obtain an adequate quantity and quality of RNA, so sequencing was not performed. For PB and JPPs, a total of 21,424 and 22,953 transcribed genes were obtained, with a mapping rate of 79 ± 3% and 78 ± 3%, respectively. Of these transcribed genes, an important part still has no nominal attribution, according to the referenced NCBI and Ensembl porcine genome databases. Therefore, the number of genes with useful attribution was, respectively, 15,509 and 16,197. The expression profile was explored with a multi-dimensional scaling plot (MDS plot) for each tissue, based on count data normalized via variance stabilized transformation. In this plot, samples are positioned according to the statistical distance of their expression profiles, showing no clear separation based on dietary group (Figure 3).

Differential expression analysis revealed that in PB samples at T1 two genes, pancreatic trypsin inhibitor (*PTI*) (Log2FC = 5.0, lgfSE = 1.1, *p* adj < 0.01) and PIGY Upstream Reading Frame (*PYURF*) were DE in favor of the NU group; the latter was also DE in JPPs, in favor of NU (Log2FC = 22.7, lgfSE = 2.9, *p* adj < 0.01). At T2, no DE genes were found for PB samples, whereas in the JPPs of NU pigs, there was a higher expression of *REG3G* compared to CO (Log2FC = 5.4, lgfSE = 1.1, *p* adj < 0.01).

The enrichment analysis conducted using the Gene Set Enrichment revealed the upregulation of 24 gene sets for CO, and 1 for NU at weaning (T1), in the PB samples (Table 4). Conversely, at the end of the trial (T2), 10 gene sets were enriched in the NU group against 4 in CO (Table 5).

**Figure 4:**
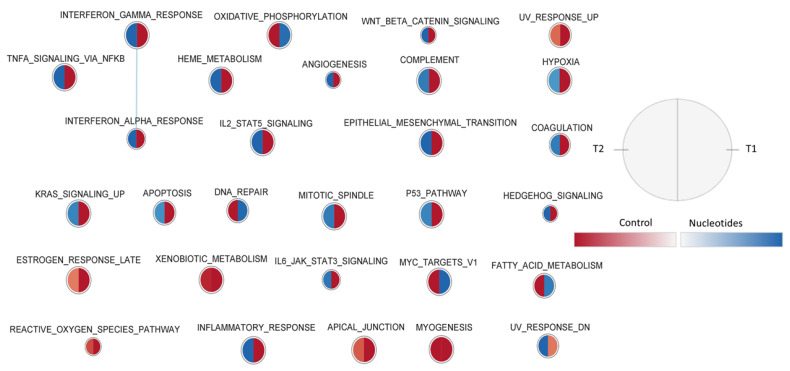
Effect of diet on peripheral blood gene sets enriched in the two sampling times, using the Hallmark collection. The nodes represent the enriched gene sets in the CO group (red) and NU group (blue). For each node, the right semicircle represents T1 and the left semicircle represents T2. The node size represents the number of genes in each gene set. The threshold for node insertion was FDR q-value < 0.05. The nodes were joined if the overlap coefficient was ≥0.375.

Figure 4 presents the effect of the diet on peripheral blood gene sets enriched in the two sampling times, using the Hallmark collection. In general, at weaning, many typical blood sets (haeme metabolism, coagulation, angiogenesis, complement, anoxia) related to cell differentiation and inflammatory response (response to interferon-alpha and gamma, signal through TNF, IL2, etc.) were dominant in the CO group, and the response was reversed 12 days after weaning. Some gene sets such as those related to the response to external organisms and to the reactive oxygen species were enriched with over-regulated genes in the CO group.

**Figure 5:**
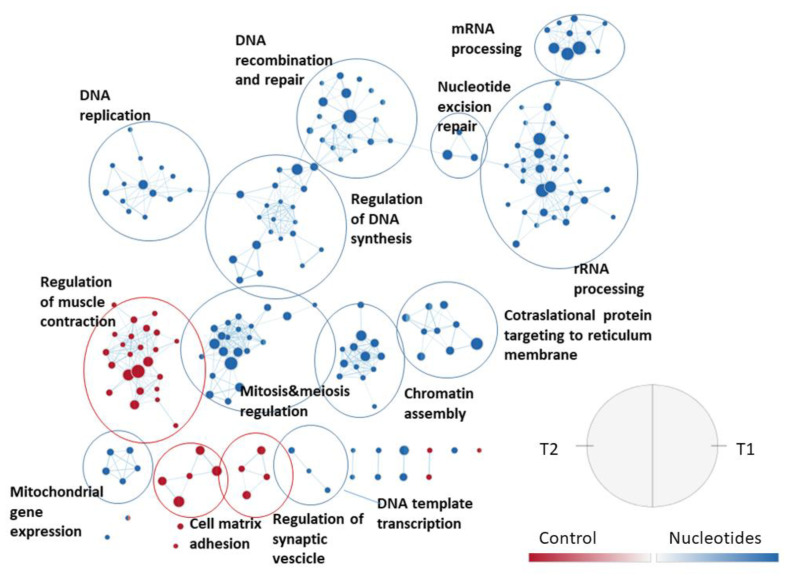
Effect of the diet on jejunal Peyer’s patches gene sets enriched in the two sampling times, using the collection of biological processes according to Gene Ontology. The nodes represent the enriched gene sets in the CO group (red) and NU group (blue). For each node, the right semicircle represents T1 and the left semicircle represents T2. The node size represents the number of genes in each gene set. The threshold for node insertion was a FDR q-value < 0.001. The nodes were joined if the overlap coefficient was ≥0.5.

For the JPPs, a more articulated gene set collection was used, which considers the biological processes detailed by Gene Ontology and includes 7530 sets. With this collection, 62 enriched gene sets were observed in T1 in the CO group and 92 in the NU group, respectively. At weaning, 445 gene sets were enriched for CO and 341 for NU. The effect of the diet on the enriched gene sets is shown in Figure 5. In this figure, the names of the gene sets are abbreviated, while Supplementary Figure S1 shows the full names. The combined representation shows that in general, the response is consistent in the two time-points. NU supplementation enriched a rather large set of genes involved in cell replication, epigenetic regulation, DNA, messenger RNA, mitochondrial RNA, and protein synthesis. On the other hand, in the CO group, there was an activation of genes related to the structuring of JPPs (junctions and cell-matrix), the organization of local smooth muscles, and neuronal control through synaptic vesicles.

**Figure 6:**
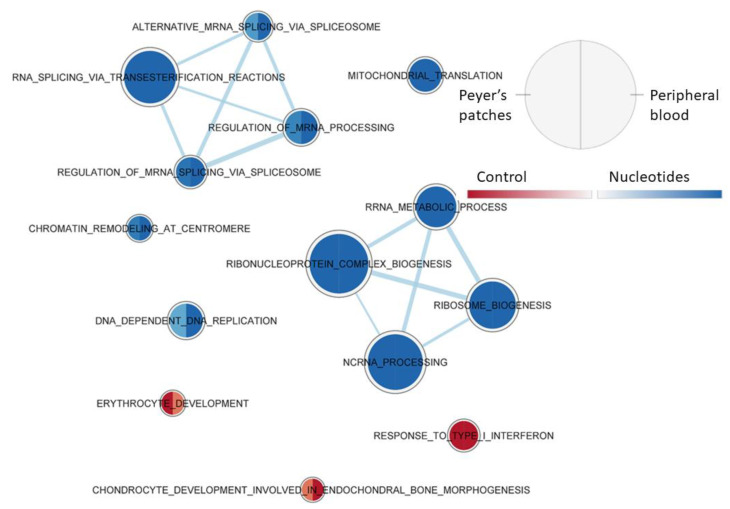
Effect of diet on PB and JPPs gene sets enriched at weaning (T1), using the collection of biological processes according to GO. The nodes represent the enriched gene sets in the CO group (red) and NU group (blue). For each node, the right semicircle represents the JPPs and the left semicircle represents PB. The node size represents the number of genes in each gene set. The threshold for node insertion was an FDR q-value < 0.001. The nodes were joined if the overlap coefficient was ≥0.5.

Figure 6 shows the effect of the diet on the gene set enrichment in the two types of tissue at the time of weaning. This showed that in the samples collected at weaning in the NU group, the gene sets involved in the processes of rRNA and mRNA (including its maturation) and chromatin were enriched, compared to CO, whatever the tissue. On the other hand, for the CO group, there was a constant activation of two types of gene set associated with the type 1 response to interferon.

In the case of the samples collected at the end of the trial, there was no equal response in the two tissues, although many gene sets had been differently involved. Consequently, Supplementary Figure S2 reports only the graphical visualization of the different responses in the two tissues at T2.

## 4. Discussion

This study investigated the effect of oral administration of nucleotides before and after weaning to piglets. Even though nucleotides did not affect the growth performances, they could explicate a positive effect on the microbiota and on the immunological maturation of the GI tract, by increasing the abundance of bacterial taxa associated with age and by increasing the proliferative activity of the JPPs.

Nucleotides represent essential compounds involved in several biological processes, as they are the building blocks of RNA, DNA, and ATP. Tissues with high cellular turnover, such as intestinal epithelium and lymphoid tissues, have a higher need for nucleotides. Additionally, in a stressful situation like weaning, a higher intake of nucleotides could be beneficial by enhancing the immune response and reducing the intestinal inflammation associated with it. The effects of nucleotide supplementation on growth performance are heterogeneous. Several studies reported no effect on growth performance [23,24,25], in agreement with our results. On the other hand, in a study by Jang and Kim (2019) [26], a supplementation of 50 and 150 mg/kg of nucleotides to newly weaned pigs increased ADG in the first week post-weaning. In another study, Perricone et al. (2020) [27] found that a higher dose (0.8 g) of nucleotide in post-weaning increases ADG, BW, and FI. These studies were mostly focused on the post-weaning phase, none of these investigated the effect starting from the suckling phase. In this context, the effect of nucleotide supply on growth performance could be related to dose, time of administration, and supplementation method (in the feed or in oral solution).

The values of the blood parameters fall within the range commonly observed for weaned piglets [28]. However, piglets in the NU group had a higher level of hematocrit and hemoglobin than piglets in the CO group. Revilla et al. (2019) [29] found a favorable correlation between the hematocrit and hemoglobin values at weaning and the piglet robustness index, concerning the first 7 weeks after weaning. Robustness is defined as the ability of an animal to maintain a certain phenotype regardless of the characteristics of the external environment [30]. This is associated with resilience, defined as the ability to cope with environmental disturbances and to quickly return to the “pre-challenge” state [29,30]. Furthermore, a positive relationship was observed between the hemoglobin and hematocrit values and daily weight gain in the first 3 weeks post-weaning [31]. In particular, a 1 g increase in hemoglobin per dL of blood would correspond to an increase in weight gain of 17 g per day [31]. In the present study, an absence of interaction between diet and age on hematocrit values indicates that the effect of supplementation on the hematocrit persists after twelve days of feeding with pre-started diets. The litters of origin were the same and the feed used was identical for the two groups and well supplemented for iron, thus dietary iron could not have been a negative factor affecting the iron state of pigs. Therefore, it can be assumed that the observed differences were associated with a direct early effect of the nucleotide supplementation and are attributable to other factors, such as the general state of health and body hydration. Conversely, no effect of nucleotides was seen on the hematocrit of piglets when given in weaning [4], indicating that starting in the suckling period with more immature pigs is relevant.

Studies involving the effect of nucleotides on microbial profile are limited. There are no studies investigating the effect of nucleotides using 16S amplicon sequencing on pig microbiota, a study by Wu et al. (2018) [7] on poultry found that nucleotides increased the alpha diversity indices (Chao1 and Shannon) in intestinal contents. However, in the present study, no effect on alpha diversity was seen. In addition, in the present study, no effect of nucleotides was evidenced for the beta diversity and limited differences were found in the abundancy of taxa. Wu et al. (2018) [7] found a higher abundance of *Lactobacillus* and a decrease in bacteria from *Blautia* and *Ruminiclostridium_5* genera with dietary yeast nucleotide supplementation in specific pathogen-free chickens. In the present study, a higher abundance of Campylobacteraceae and a decrease in Streptococcaceae was associated with the nucleotide supply starting from 10 days of age. The Campylobacteraceae family belongs to the Epsilonbacteraeota phylum that was consequently also more abundant in NU. In the gastrointestinal tract of the piglet, the Campylobacteraceae family is made up of both pathogenic (e.g., *Campylobacter jejuni*) and commensal bacteria [32]. Furthermore, this taxon is generally associated with an increase in the maturation of piglet gut microbiota as its abundancy increases with age in the post-weaning phases [32]; in addition, a greater abundance of the Campylobacteraceae family in the caecal mucosa has been associated with better feeding efficiency in growing pigs [33]. Streptococcaceae, on the other hand, are a predominant taxon in the gut microbiota of piglets in the suckling phase [34]. Therefore, the lower abundance of Streptococcaceae and the greater abundance of Campylobacteraceae in the NU pigs at weaning may indicate that the intestinal microbiota of piglets receiving the nucleotide supply was already more mature than that of the control. Furthermore the ability of adhering to the intestinal mucosa, recognized in several strains belonging to Campylobacteraceae [35], could be a factor associated with the increased immune response observed with NU. Nevertheless, more studies are needed to better understand the role of commensal Campyloabaceraceae in young pigs.

The differences seen in the presence of some bacteria associated with the addition or not of the nucleotide-based product could be related to a different activation of the local immune system [3,4]. Furthermore, all the pigs in the trial were stimulated at 18 d of age with anti-ETEC vaccination, and for this purpose we selected ETEC-susceptible pigs. To investigate the effect of nucleotides supplementation on gene expression profile, we used a global transcriptomic approach to evidence mRNA changes in both PB and JPPs. At weaning, the transcriptomic profile of the PB samples evidenced a higher expression of *PTI* and *PYURF* genes in the NU group, and the latter was also DE in JPPs samples. *PYURF* encodes the upstream open reading frame of the bicistronic transcript that encodes for the biosynthesis of a phosphatidylinositol glycan anchor protein (PIGY) [36]. In pigs, this gene is located in the chromosome 8, in a QTL that was associated with porcine hematocrit variation [37]. Interestingly, hematocrit values were also positively associated with NU. However, generally, the *PYURF* expression was low and limited to some subjects, thus further studies are required to clarify this aspect.

*PTI* is a gene for which there is not a true homolog in humans, coding for a pancreatic trypsin inhibitor, long known for pigs [38]. In the pancreas, it has a protective action, while in the blood it could have relevance as an antifibrinolytic factor on the homology of the molecule of bovine origin [39]. Regulation of the activation of *PTI* has not been studied previously, even though there is increasing interest in medicine regarding this kind of protein. The upregulation of *PTI* in the present case may be associated with the antifibrinolytic effect and in general with the slightly different percentage of blood volume occupied by erythrocytes. However, using a rapid visualization of the plot of data, no association between *PTI* expression and hematocrit was seen.

At T2, no DE gene in the PB was found, and conversely in JPPs, the nucleotide supplementation increased the *REG3G* expression. The homologous protein transcribed from this gene, with known antimicrobial action, is typical of the intestinal epithelium and its activation can be stimulated by both beneficial microorganisms, such as *Lactobacillus plantarum* [40] or pathogens (like *E. coli* K88, [41]). In our case, in healthy subjects, it could be an indicator of increased activation of epithelial defense. In fact, it should be underlined that the pigs were vaccinated with two non-pathogenic *E. coli* strains bearing both F4 and F18 fimbria, and that ETEC-susceptible healthy pigs also previously showed higher expression of *REG3G* [42].

The enrichment analysis showed that nucleotide supplementation induced a greater activation of the replicative and productive functions of the JPPs that was consistent in the two sampling times. Lymphocytes are the main cellular component of Peyer’s patches; thus, it can be assumed that the activation of these genes concerns mainly this class of cells. The intensification of lymphocytic replication in the jejunum and ileal plaques is a typical indicator of their maturation with age [43]. It would be tempting to state that this greater activation was maintained by the early provision of the dietary addition of the nucleotide-based product, in agreement with the high mitotic index observed in ileal mucosa of weaned pigs [23]. In fact, lymphocytes have a limited ability to produce nucleotides to optimize their proliferation and maturation [44]. Their higher multiplicative and productive activity in supplemented pigs may also explain the better ability of Peyer’s patches to produce more inflammatory cytokines of post-weaning pigs reared in poor environmental condition [45] and the increased IgA production [4]. Conversely, this mechanism was not more active in standard rearing conditions [27], where Peyer’s Patches activation of inflammatory cytokines was not seen with the addition of nucleotides, while this addition promoted growth response of piglets.

On the other hand, in the CO group, a higher activation of genes related to JPPs structuring (junctions and cell-matrix), organization of local smooth muscles, and neuronal control through synaptic vesicles, was seen. The effects were consistent in both the time-points considered. The presence of groups of genes typical of other tissues is not surprising because overall JPPs are an aggregate of complex cellular structures in which cells of natural endothelial and keratinocytic types also play a role, which also gives them a circulatory function and a structural consistency. In particular, the porous nature of the local lamina propria assures the migration of antigens from the surface epithelium and M cells to the dendritic cells in the sub-epithelial dome [46,47]. Interestingly, cytokeratin (18) was proposed as a marker of M-cells in porcine Peyer’s patches, presumably contributing to their specific shape [48]. In general, the structural cells supporting Peyer’s patches received scarce research attention. It is possible that due to the increased activation of lymphocytes in the treated pig group, relatively more mRNA was present in the control related to these structural genes.

For PB samples, the time-dependent different presence of enriched gene sets in the two treatment groups apparently contrasts with the dietary effect in the absence of a significant interaction with time, observed for some of the blood cell counts. For instance, the enrichment of gene sets associated with heme metabolism, coagulation, angiogenesis, and anoxia in the immediate pre-weaning in the control group may be explained by the need to attain a higher volume for the blood cells in PB like the one observed in piglets supplemented with the nucleotide-based product. Conversely, the enrichment of the same gene sets in samples obtained from supplemented pigs on day 12 post-weaning, in the presence of a relatively higher hematocrit, suggests that these pigs were further activating their erythropoiesis to improve their oxidative status. Taken together, these data may indicate that nucleotides delayed the need for oxygen support for the oxygen-demanding growth in the post-weaning.

No other direct evidence of that has been reported, but in neonatal rats, nucleotides increased the concentration of 2,3-diphosphoglycerate in erythrocytes [49], possibly improving the exchange of oxygen in tissues, because hemoglobin has more affinity for this molecule compared to oxygen. Moreover, the nucleotide supplementation improved the unsaturation index of red blood cell phospholipids in human neonate [50] and weanling rat [51], with a possible impact on their regulation of the metabolic activity of these cells. This, however, was not seen in preterm babies [52]. Taken together, these observations in other species, indicating a possible better degree of systemic oxidation and a reduced need of activation of hematopoiesis with nucleotide supplementation, can explain the reduced activation of genes related to heme metabolism in the immediate pre-weaning. However, it also contrasts with the constantly higher hemoglobin content and blood cell density with the supplementation of nucleotides that needs other explanations. Finally, concerning hematocrit variations, we calculated the simple correlations of this parameter with the expressions of gene in PB (data not shown), and it is interesting to report that the best correlation across time and feeding groups was seen for Junctional Cadherin 5 Associated (*JCAD*) (r = +0.591). The same was also with hemoglobin (r = +0.647). Scarce information is available on *JCAD*. However, the protein coded by *JCAD* was located in blood endothelial cell–cell junctions from human tissues [53] and mutations of that gene were associated with atherosclerosis and hypertension [54]. Thus, it is tempting to propose this gene as an indicator of blood erythrocyte density and to consider it for further studies.

A time-dependent effect of the diet on PB transcriptome was also seen on several gene sets related to inflammatory response (primarily INTERFERON_ALPHA_RESPONSE, INTERFERON_GAMMA_RESPONSE, TNFA_SIGNALING_VIA_NFKB). In the immediate pre-weaning, in the NU group, a lower initial activation of these gene sets was seen compared to the CO group, and this agreed also with the reduced enrichment of the set of the other collection, RESPONSE_TO_ TYPE_I_INTERFERON, observed for both PB and JPP (Figure 5). For PB, no effect of the diet was seen on the number and relative partition of different classes of leucocytes, thus it can be assumed that the effect on transcriptome was related to the modulation of some of them. This could be associated with the increased activity of JPP and to the ability to control the translocation of microorganisms to the whole-body system in NU compared to CO, such as increased production of IgA [4]. On the contrary, the increased presence of enrichment sets on day 12 post-weaning in NU could depend on a shift in the diffuse immune system related to the continuing maturation progress. However, the overall regulation seems quite complex, as indicated conversely by the divergent response of transcriptome between PB and JPPs.

Overall, the transcriptomic profile showed an intense proliferative activity in the JPPs of piglets of the NU group, exhibited by the activation of a series of gene sets, ranging from epigenetic response to transcriptional regulation. This could be a sign of a more advanced state of maturation, which may have been favored by the supplementation of nucleotides.

## 5. Conclusions

Nucleotide supplementation did not influence the growth performances but could have favored an early maturation of the gastrointestinal microbiota by increasing the abundance of bacterial taxa associated with older pigs (Campylobacteraceae). For the transcriptomic profile, a complex, time- and tissue-dependent effect was seen. In fact, the nucleotide supplementation induced a higher proliferative activity in the JPPs, possibly reduced the inflammation in the immediate pre-weaning, and increased the erythropoietic activity in the post-weaning in the PB. Further studies are needed in order to investigate the effect of nucleotides on the pig microbiota structure and the immune maturation of the gastrointestinal tract.

## Figures and Tables

**Table 1:** Ingredients and calculated composition of the feed expressed on as fed.

Ingredients	Units	Content
Bakery former food	%	20.00
Barley	%	15.00
Soybean Protein Concentrate	%	13.50
Wheat, soft	%	12.20
Maize	%	11.75
Whey, sweet, dehydrated, skimmed	%	9.00
Wheat middlings	%	5.00
Spray dried porcine plasma	%	3.00
Lard	%	2.00
Beet pulp, dehydrated	%	1.50
Dicalcium phosphate anhydrous	%	1.40
Dextrose	%	1.20
Medium chain free fatty acid mixture	%	1.00
Organic acid mixture	%	1.00
l-Lysine HCl	%	0.55
Calcium carbonate	%	0.53
Sodium chloride	%	0.30
Vitamin and trace mineral mixture ^1^	%	0.30
dl-Methionine	%	0.28
l-Threonine	%	0.28
l-Valine	%	0.11
l-Tryptophan	%	0.10
Calculated values ^2^		
Metabolizable energy	Kcal/kg	3340
Crude Protein	%	18.00
Crude Fat	%	6.44
Crude Fibre	%	2.75
Ash	%	5.71
Lysine	%	1.34
Cysteine	%	0.30
Methionine	%	0.51
Threonine	%	0.95
Tryptophan	%	0.29
Valine	%	0.99

^1^ Provided per kg of diet: vitamin A (retinyl acetate): 7500 IU; vitamin D3 (cholecalciferol): 1000 IU; vitamin E (dl-α_tocopheryl acetate): 80 mg; vitamin K (menadione sodium bisulfite): 1 mg; riboflavin, 2.3 mg; calcium-d-pantothenate: 9.0 mg; niacin: 17.5 mg; pyridoxine hydrochloride: 1 mg; folic acid: 0.5 mg; biotin: 0.10 mg; thiamine: 1.45 mg; vitamin B12: 15 μg; ferrous sulphate monohydrate: 302.8 mg; zinc oxide: 118.2 mg; manganous sulphate monohydrate: 18.48; copper sulphate: 347.6; sodium selenite: 0.55 mg; potassium iodide: 1.31 mg. ^2^ The values were estimated using EvaPig^®^ software (Noblet et al., 2008) with information from the INRA-AFZ tables of feedstuff composition. Noblet, J., Valancogne, A., Tran, G., Ajinomoto Eurolysine sas, 2008. EvaPig^®^ [1.0. 1.4]. Computer program.

**Table 2:** Performance data (weaning at d 26; CO = control group; NU = nucleotides group; BW = body weight, ADG = average daily gain, FI = feed intake, F:G = feed to gain ratio).

Item	CO	NU	SEM	*p*-Value
BW, d 10, kg	3.49	3.47	0.09	0.872
BW, d 21, kg	6.38	6.22	0.14	0.409
BW, d 26, kg	7.25	7.09	0.17	0.450
ADG, d 10-d21, g	263.2	250.1	7.8	0.231
ADG, d 10-d26, g	235.2	226.4	7.2	0.381
BW, d 38, kg	9.55	9.38	0.20	0.559
ADG, d 10-d38, g	216.5	211.0	5.2	0.448
ADG, d 28-d38, g	190.1	188.1	7.5	0.849
FI, d 26-d38, g	222.2	216.4	5.5	0.453
F:G, d 26-d39	1.33	1.20	0.08	0.225
Days with diarrea, n	0.53	0.71	0.17	0.433

**Table 3:** Blood data obtained on fresh samples, for subjects sacrificed at weaning and 12 days later.

Item ^1^	Diet		SEM	Sampling Time		SEM	*p*-Value		
	CO	NU		Weaning	d 12 Post-Weaning		Diet ^1^	Sex	Sampling Time ^2^
RBC, M/µL	6.52	6.73	0.11	6.40	6.86	0.12	0.114	0.844	0.013
HGB, g/dL	11.6	12.0	0.2	11.7	11.9	0.2	0.032	0.256	0.517
HCT, %	35.4	36.9	0.6	35.2	37.1	0.6	0.026	0.354	0.037
MCV, fL	54.5	55.1	0.6	55.3	54.3	0.7	0.469	0.196	0.327
MCH, pg	17.9	18.0	0.2	18.4	17.4	0.2	0.736	0.182	0.003
MCHC, g/dL	32.9	32.7	0.2	33.5	32.1	0.2	0.403	0.776	0.0001
RDW, %	24.4	23.2	0.6	23.5	24.0	0.7	0.102	0.203	0.624
PLT, K/µL	507	537	33	580	464	35	0.434	0.955	0.028
WBC, K/µL	13.8	13.7	0.8	11.4	16.0	0.85	0.897	0.012	0.0004
Neutrophils, K/µL	6.87	7.09	0.605	6.94	7.03	0.64	0.757	0.432	0.926
Lymphocytes, K/µL	5.43	4.99	0.62	3.22	7.20	0.65	0.545	0.023	0.0001
Monocytes, K/µL	1.15	1.26	0.15	1.05	1.36	0.16	0.559	0.496	0.209
Eosinophils, K/µL	0.13	0.11	0.040	0	0.25	0.045	0.653	0.365	<0.0001
Basophils, K/µL	0.19	0.18	0.040	0.15	0.22	0.045	0.744	0.042	0.294
Neutrophils, %	51.9	51.6	2.4	59.7	43.8	2.56	0.901	0.543	<0.0001
Lymphocytes, %	36.7	37.1	3.0	29.5	44.3	3.14	0.929	0.486	0.002
Monocytes, %	8.95	9.29	0.995	9.33	8.91	1.06	0.774	0.568	0.793
Eosinophils, %	0.97	0.71	0.28	0	1.75	0.295	0.434	0.155	0.0001
Basophils, %	1.43	1.23	0.22	1.37	1.29	0.23	0.432	0.225	0.830

^1^ WBC—white blood cells; RBC—red blood cells; HGB—hemoglobin; HCT—hematocrit; MCV—mean corpuscular volume; MCH—mean corpuscular hemoglobin; MCHC—mean corpuscular hemoglobin concentration; RDW—red blood cell distribution width; MPV—mean platelet volume. ^2^ The interaction between diet and sampling time was not statistically significant, and thus was excluded from the statistical model.

**Table 4:** Significantly enriched gene groups in PB, respectively for CO and NU groups, according to the Hallmark collection, at weaning (T1).

Gene Set Name	Total Number of Genes	Normalized Standard Error	*p*-Value	FDR q-Value
CO Group				
HEME_METABOLISM	185	2.922	0.000	0.000
INTERFERON_ALPHA_RESPONSE	87	2.820	0.000	0.000
INTERFERON_GAMMA_RESPONSE	177	2.672	0.000	0.000
IL6_JAK_STAT3_SIGNALING	80	2.106	0.000	0.000
TNFA_SIGNALING_VIA_NFKB	192	1.903	0.000	0.000
MYOGENESIS	188	1.863	0.000	0.001
P53_PATHWAY	187	1.772	0.000	0.002
KRAS_SIGNALING_UP	183	1.772	0.000	0.002
INFLAMMATORY_RESPONSE	188	1.767	0.000	0.001
MITOTIC_SPINDLE	198	1.759	0.000	0.001
ANGIOGENESIS	31	1.696	0.002	0.003
COMPLEMENT	185	1.678	0.000	0.004
ESTROGEN_RESPONSE_LATE	174	1.646	0.000	0.005
XENOBIOTIC_METABOLISM	169	1.585	0.000	0.009
APOPTOSIS	154	1.571	0.001	0.010
APICAL_JUNCTION	184	1.559	0.001	0.011
IL2_STAT5_SIGNALING	191	1.544	0.001	0.013
COAGULATION	116	1.530	0.006	0.014
WNT_BETA_CATENIN_SIGNALING	39	1.524	0.025	0.015
EPITHELIAL_MESENCHYMAL_TRANSITION	185	1.508	0.001	0.017
REACTIVE_OXYGEN_SPECIES_PATHWAY	49	1.460	0.034	0.026
HYPOXIA	186	1.429	0.006	0.033
HEDGEHOG_SIGNALING	32	1.422	0.055	0.033
UV_RESPONSE_UP	145	1.405	0.009	0.036
NU Group				
MYC_TARGETS_V1	193	−2.469	0	0

**Table 5:** Significantly enriched gene groups in PB, respectively, for CO and NU groups, according to Table 2.

Gene Set Name	Total Number of Genes	Normalized Standard Error	*p*-Value	FDR q-Value
CO group				
OXIDATIVE_ PHOSPHORYLATION	167	2.826	0.000	0.000
MYC_TARGETS_V1	193	2.408	0.000	0.000
DNA_REPAIR	143	2.159	0.000	0.000
FATTY_ACID_ METABOLISM	140	1.999	0.000	0.000
NU group				
EPITHELIAL_ MESENCHYMAL_ TRANSITION	185	−1.823	0.000	0.014
INTERFERON_ ALPHA_RESPONSE	87	−1.720	0.000	0.017
UV_RESPONSE_DN	136	−1.696	0.000	0.015
TNFA_SIGNALING_ VIA_NFKB	192	−1.665	0.000	0.016
HEME_METABOLISM	185	−1.664	0.002	0.013
INTERFERON_ GAMMA_RESPONSE	177	−1.550	0.002	0.031
ANGIOGENESIS	31	−1.526	0.039	0.032
HEDGEHOG_ SIGNALING	32	−1.516	0.029	0.032
INFLAMMATORY_ RESPONSE	188	−1.456	0.004	0.048

## Data Availability

The raw reads obtained from the 16s and mRNA sequencing are publicly available at the NCBI Sequence Read Archive (SRA) under the accession numbers SUB8665787 and SUB8684880, respectively.

## Supplementary Materials

The following are available online at https://www.mdpi.com/article/10.3390/ani11061489/s1, Figure S1: Effect of the diet on jejunal Peyer’s patches gene sets enriched in the two sampling times, with the full name of each enriched gene set. The nodes represent the enriched gene sets in the CO group (red) and NU group (blue). For each node, the right semicircle represents T1 and the left semicircle represents T2. The node size represents the number of genes in each gene set. The threshold for node insertion was a FDR q-value < 0.001. The nodes were joined if the overlap coefficient was ≥0.5. Figure S2. Effect of diet on PB and JPPs gene sets enriched at 12 day post-weaning (T2), using the collection of biological processes according to GO. The nodes represent the enriched gene sets in the CO group (red) and NU group (blue). For each node, the right semicircle represents the JPPs and the left semicircle represents PB. The node size represents the number of genes in each gene set. The threshold for node insertion was an FDR q-value < 0.001. The nodes were joined if the overlap coefficient was ≥0.5.

## References

[1] Van Buren C.T., Rudolph F. (1997). Dietary Nucleotides: A Conditional Requirement. Nutrition.

[2] Uauy R., Quan R., Gil A. (1994). Role of Nucleotides in Intestinal Development and Repair: Implications for Infant Nutrition. J. Nutr..

[3] Grimble G.K., Westwood O.M. (2001). Nucleotides as Immunomodulators in Clinical Nutrition. Curr. Opin. Clin. Nutr. Metab. Care.

[4] Sauer N., Mosenthin R., Bauer E. (2011). The Role of Dietary Nucleotides in Single-Stomached Animals. Nutr. Res. Rev..

[5] Mateo C.D. (2005). Aspects of Nucleotide Nutrition in Pigs. Ph.D. Thesis.

[6] Mateo C.D., Peters D.N., Stein H.H. (2004). Nucleotides in Sow Colostrum and Milk at Different Stages of Lactation. J. Anim. Sci..

[7] Wu C., Yang Z., Song C., Liang C., Li H., Chen W., Lin W., Xie Q. (2018). Effects of Dietary Yeast Nucleotides Supplementation on Intestinal Barrier Function, Intestinal Microbiota, and Humoral Immunity in Specific Pathogen-Free Chickens. Poult. Sci..

[8] Fairbrother J.M., Nadeau É., Bélanger L., Tremblay C.-L., Tremblay D., Brunelle M., Wolf R., Hellmann K., Hidalgo Á. (2017). Immunogenicity and Protective Efficacy of a Single-Dose Live Non-Pathogenic Escherichia Coli Oral Vaccine against F4-Positive Enterotoxigenic Escherichia Coli Challenge in Pigs. Vaccine.

[9] Jørgensen C.B., Cirera S., Anderson S.I., Archibald A.L., Raudsepp T., Chowdhary B., Edfors-Lilja I., Andersson L., Fredholm M. (2003). Linkage and Comparative Mapping of the Locus Controlling Susceptibility towards E. Coli F4ab/Ac Diarrhoea in Pigs. Cytogenet. Genome Res..

[10] Butler J.E., Santiago-Mateo K., Wertz N., Sun X., Sinkora M., Francis D.L. (2016). Antibody Repertoire Development in Fetal and Neonatal Piglets. XXIV. Hypothesis: The Ileal Peyer Patches (IPP) Are the Major Source of Primary, Undiversified IgA Antibodies in Newborn Piglets. Dev. Comp. Immunol..

[11] Takahashi S., Tomita J., Nishioka K., Hisada T., Nishijima M. (2014). Development of a Prokaryotic Universal Primer for Simultaneous Analysis of Bacteria and Archaea Using Next-Generation Sequencing. PLoS ONE.

[12] Callahan B.J., McMurdie P.J., Rosen M.J., Han A.W., Johnson A.J.A., Holmes S.P. (2016). DADA2: High-Resolution Sample Inference from Illumina Amplicon Data. Nat. Methods.

[13] McMurdie P.J., Holmes S. (2013). Phyloseq: An R Package for Reproducible Interactive Analysis and Graphics of Microbiome Census Data. PLoS ONE.

[14] Dixon P. (2003). VEGAN, a Package of R Functions for Community Ecology. J. Veg. Sci..

[15] Bates D., Mächler M., Bolker B., Walker S. (2015). Fitting Linear Mixed-Effects Models Using Lme4. J. Stat. Softw..

[16] Bolger A.M., Lohse M., Usadel B. (2014). Trimmomatic: A Flexible Trimmer for Illumina Sequence Data. Bioinform. Oxf. Engl..

[17] Patro R., Duggal G., Kingsford C. (2015). Salmon: Accurate, Versatile and Ultrafast Quantification from RNA-Seq Data Using Lightweight-Alignment. bioRxiv.

[18] Love M.I., Huber W., Anders S. (2014). Moderated Estimation of Fold Change and Dispersion for RNA-Seq Data with DESeq2. Genome Biol..

[19] Bild A., Febbo P.G. (2005). Application of a Priori Established Gene Sets to Discover Biologically Important Differential Expression in Microarray Data. Proc. Natl. Acad. Sci. USA.

[20] Liberzon A., Birger C., Thorvaldsdóttir H., Ghandi M., Mesirov J.P., Tamayo P. (2015). The Molecular Signatures Database (MSigDB) Hallmark Gene Set Collection. Cell Syst..

[21] Merico D., Isserlin R., Stueker O., Emili A., Bader G.D. (2010). Enrichment Map: A Network-Based Method for Gene-Set Enrichment Visualization and Interpretation. PLoS ONE.

[22] Shannon P., Markiel A., Ozier O., Baliga N.S., Wang J.T., Ramage D., Amin N., Schwikowski B., Ideker T. (2003). Cytoscape: A Software Environment for Integrated Models of Biomolecular Interaction Networks. Genome Res..

[23] Domeneghini C., Di Giancamillo A., Savoini G., Paratte R., Bontempo V., Dell’Orto V. (2004). Structural Patterns of Swine Ileal Mucosa Following L-Glutamine and Nucleotide Administration during the Weaning Period. An Histochemical and Histometrical Study. Histol. Histopathol..

[24] Lee D.N., Liu S.R., Chen Y.T., Wang R.C., Lin S.Y., Weng C.F. (2007). Effects of Diets Supplemented with Organic Acids and Nucleotides on Growth, Immune Responses and Digestive Tract Development in Weaned Pigs. J. Anim. Physiol. Anim. Nutr..

[25] Martinez-Puig D., Manzanilla E.G., Morales J., Borda E., Pérez J.F., Piñeiro C., Chetrit C. (2007). Dietary Nucleotide Supplementation Reduces Occurrence of Diarrhoea in Early Weaned Pigs. Livest. Sci..

[26] Jang K.B., Kim S.W. (2019). Supplemental Effects of Dietary Nucleotides on Intestinal Health and Growth Performance of Newly Weaned Pigs. J. Anim. Sci..

[27] Perricone V., Comi M., Bontempo V., Lecchi C., Ceciliani F., Crestani M., Ferrari A., Savoini G., Agazzi A. (2020). Effects of Nucleotides Administration on Growth Performance and Immune Response of Post-Weaning Piglets. Ital. J. Anim. Sci..

[28] Perri A.M., O’Sullivan T.L., Harding J.C.S., Wood R.D., Friendship R.M. (2017). Hematology and Biochemistry Reference Intervals for Ontario Commercial Nursing Pigs Close to the Time of Weaning. Can. Vet. J..

[29] Revilla M., Friggens N.C., Broudiscou L.P., Lemonnier G., Blanc F., Ravon L., Mercat M.J., Billon Y., Rogel-Gaillard C., Floch N.L. (2019). Towards the Quantitative Characterisation of Piglets’ Robustness to Weaning: A Modelling Approach. Animal.

[30] Colditz I.G., Hine B.C. (2016). Resilience in Farm Animals: Biology, Management, Breeding and Implications for Animal Welfare. Anim. Prod. Sci..

[31] Bhattarai S., Nielsen J.P. (2015). Association between Hematological Status at Weaning and Weight Gain Post-Weaning in Piglets. Livest. Sci..

[32] De Rodas B., Youmans B.P., Danzeisen J.L., Tran H., Johnson T.J. (2018). Microbiome Profiling of Commercial Pigs from Farrow to Finish. J. Anim. Sci..

[33] Metzler-Zebeli B.U., Lawlor P.G., Magowan E., Zebeli Q. (2018). Interactions between Metabolically Active Bacteria and Host Gene Expression at the Cecal Mucosa in Pigs of Diverging Feed Efficiency. J. Anim. Sci..

[34] Petri D., Hill J.E., Van Kessel A.G. (2010). Microbial Succession in the Gastrointestinal Tract (GIT) of the Preweaned Pig. Livest. Sci..

[35] Scanlon K.A., Cagney C., Walsh D., McNulty D., Carroll A., McNamara E.B., McDowell D.A., Duffy G. (2013). Occurrence and Characteristics of Fastidious Campylobacteraceae Species in Porcine Samples. Int. J. Food Microbiol..

[36] Bruford E.A., Braschi B., Denny P., Jones T.E.M., Seal R.L., Tweedie S. (2020). Guidelines for Human Gene Nomenclature. Nat. Genet..

[37] Ponsuksili S., Reyer H., Trakooljul N., Murani E., Wimmers K. (2016). Single- and Bayesian Multi-Marker Genome-Wide Association for Haematological Parameters in Pigs. PLoS ONE.

[38] Bartelt D.C., Shapanka R., Greene L.J. (1977). The Primary Structure of the Human Pancreatic Secretory Trypsin Inhibitor. Amino Acid Sequence of the Reduced S-Aminoethylated Protein. Arch. Biochem. Biophys..

[39] Gregorczyk I., Maślanka T. (2019). Effect of Selected Non-Steroidal Anti-Inflammatory Drugs on Activation-Induced CD25 Expression on Murine CD4+ and CD8+ T Cells: An in Vitro Study. Cent. Eur. J. Immunol..

[40] Gross G., van Der Meulen J., Snel J., van Der Meer R., Kleerebezem M., Niewold T.A., Hulst M.M., Smits M.A. (2008). Mannose-Specific Interaction of Lactobacillus Plantarum with Porcine Jejunal Epithelium. FEMS Immunol. Med. Microbiol..

[41] Trevisi P., Priori D., Jansman A.J.M., Luise D., Koopmans S.-J., Hynönen U., Palva A., van der Meulen J., Bosi P. (2018). Molecular Networks Affected by Neonatal Microbial Colonization in Porcine Jejunum, Luminally Perfused with Enterotoxigenic Escherichia Coli, F4ac Fimbria or Lactobacillus Amylovorus. PLoS ONE.

[42] Luise D., Motta V., Bertocchi M., Salvarani C., Clavenzani P., Fanelli F., Pagotto U., Bosi P., Trevisi P. (2019). Effect of Mucine 4 and Fucosyltransferase 1 Genetic Variants on Gut Homoeostasis of Growing Healthy Pigs. J. Anim. Physiol. Anim. Nutr..

[43] Pabst R., Geist M., Rothkötter H.J., Fritz F.J. (1988). Postnatal Development and Lymphocyte Production of Jejunal and Ileal Peyer’s Patches in Normal and Gnotobiotic Pigs. Immunology.

[44] Carver J.D. (1999). Dietary Nucleotides: Effects on the Immune and Gastrointestinal Systems. Acta Paediatr..

[45] Waititu S.M., Yin F., Patterson R., Yitbarek A., Rodriguez-Lecompte J.C., Nyachoti C.M. (2017). Dietary Supplementation with a Nucleotide-Rich Yeast Extract Modulates Gut Immune Response and Microflora in Weaned Pigs in Response to a Sanitary Challenge. Anim. Int. J. Anim. Biosci..

[46] Panneerselvam D., Budh D.P. (2020). Peyer Patches. StatPearls.

[47] Takeuchi T., Gonda T. (2004). Distribution of the Pores of Epithelial Basement Membrane in the Rat Small Intestine. J. Vet. Med. Sci..

[48] Gebert A., Rothkötter H.-J., Pabst R. (1994). Cytokeratin 18 Is an M-Cell Marker in Porcine Peyer’s Patches. Cell Tissue Res..

[49] Scopesi F., Verkeste C.M., Paola D., Gazzolo D., Pronzato M.A., Bruschettini P.L., Marinari U.M. (1999). Dietary Nucleotide Supplementation Raises Erythrocyte 2,3-Diphosphoglycerate Concentration in Neonatal Rats. J. Nutr..

[50] DeLucchi C., Pita M.L., Faus M.J., Molina J.A., Uauy R., Gil A. (1987). Effects of Dietary Nucleotides on the Fatty Acid Composition of Erythrocyte Membrane Lipids in Term Infants. J. Pediatr. Gastroenterol. Nutr..

[51] Boza J., Jimenez J., Faus M.J., Gil A. (1992). Influences of Postnatal Age and Dietary Nucleotides on Plasma Fatty Acids in the Weanling Rat. J. Parenter. Enter. Nutr..

[52] Axelsson I., Flodmark C.E., Räihä N., Tacconi M., Visentin M., Minoli I., Moro G., Warm A. (1997). The Influence of Dietary Nucleotides on Erythrocyte Membrane Fatty Acids and Plasma Lipids in Preterm Infants. Acta Paediatr..

[53] Shigeoka M., Arimoto S., Akashi M. (2020). JCAD Expression and Localization in Human Blood Endothelial Cells. Heliyon.

[54] Williams E.G., Stein S. (2019). JCAD: From Systems Genetics Identification to the Experimental Validation of a Coronary Artery Disease Risk Locus. Eur. Heart J..

