# Supplementary figures and images for: Investigation of Early Supplementation of Nucleotides on the Intestinal Maturation of Weaned Piglets
Source: Animals (Basel). 2021 May 21;11(6):1489. doi: 10.3390/ani11061489 (PMC8223990; doi:10.3390/ani11061489)

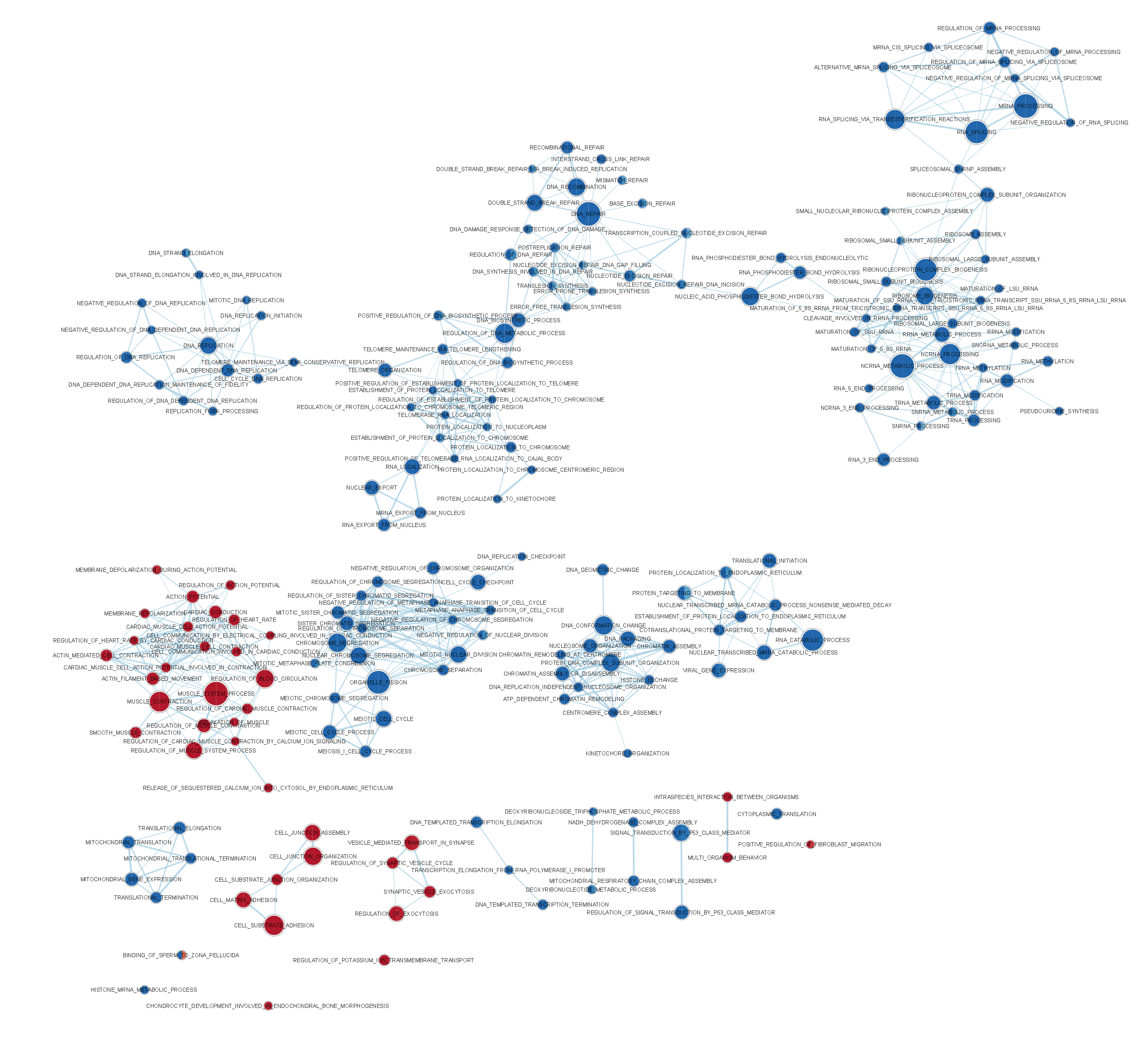

Supplement: Supplementary file 1 [file animals-11-01489-s001.zip › Supplementary Fig 1.png]

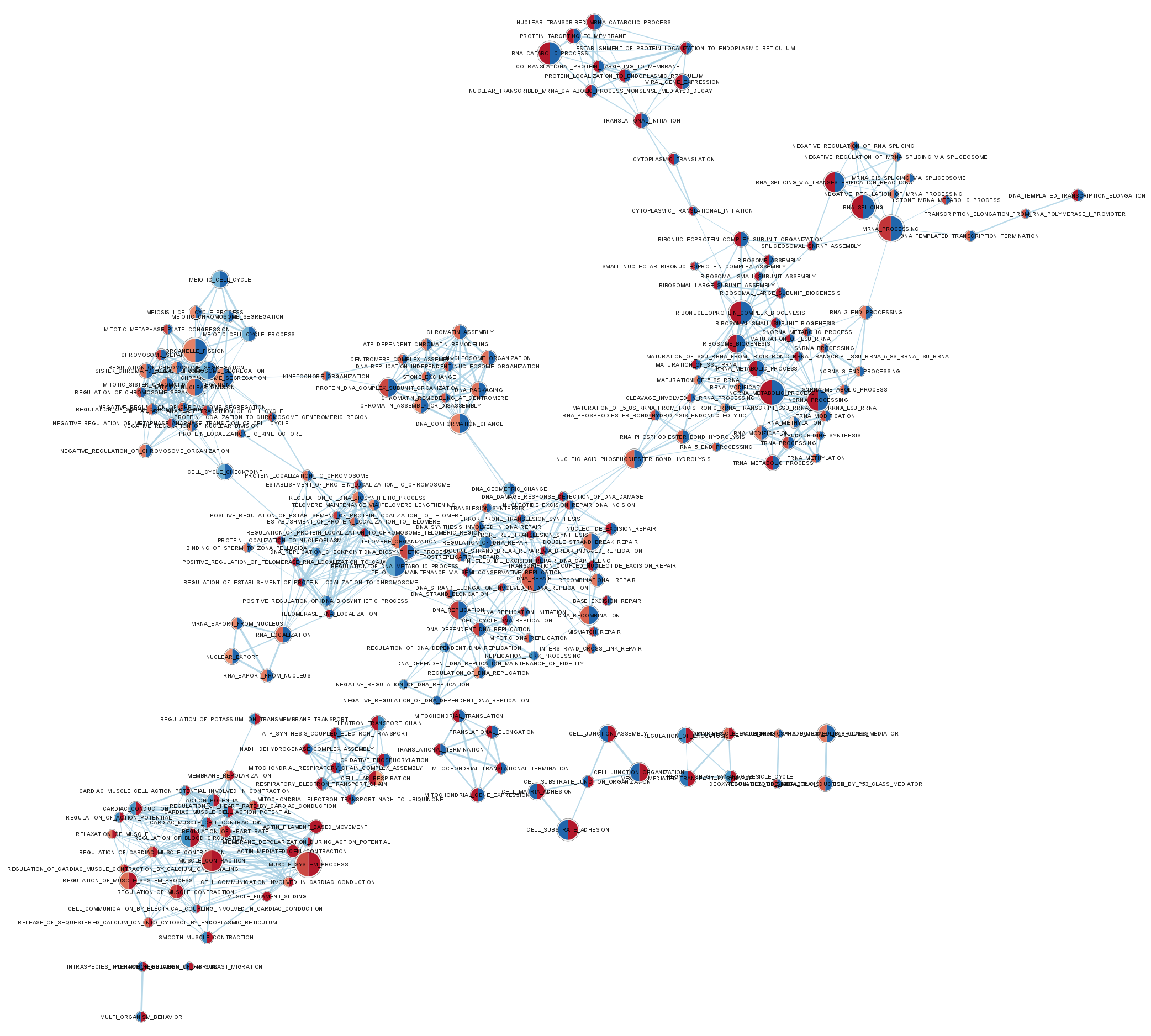

Supplement: Supplementary file 1 [file animals-11-01489-s001.zip › Supplementary Fig 2.png]
